# Complete plastomes of three endemic Mexican pine species (*Pinus* subsection *Australes*)

**DOI:** 10.1080/23802359.2017.1365637

**Published:** 2017-08-22

**Authors:** Xitlali Aguirre-Dugua, David S. Gernandt

**Affiliations:** Departamento de Botánica, Instituto de Biología, Universidad Nacional Autónoma de México, Ciudad de México, Mexico

**Keywords:** Plastome, *Pinus*, Hyb-Seq, Mexico, *Australes*, phylogenetics

## Abstract

We assembled the plastomes of *Pinus greggii*, *P. jaliscana* and *P. oocarpa* from 100 bp paired-end Illumina reads. We combined *de novo* (comparing *Velvet* and *SPAdes*) with reference-guided assembly and a final step of gap filling. *SPAdes* performed better than *Velvet* based on scaffold number (180 vs. 263) and mean length (1886 vs. 560 bp), and number of gaps (2 vs. 4). Annotations were automatically transferred from *P. taeda* NC_021440 and carefully revised by hand. Phylogenetic analysis with additional plastomes revealed very short branch lengths, supporting a rapid diversification within *Australes* and close relatedness among pines from Western Mexico.

Understanding diversification processes in *Pinus* has proven a difficult task due to their large genome with highly repetitive elements (ca. 23 Gbp; Neale et al. 2014), large effective population sizes, weak reproductive barriers and the recent origin of many of its species. Published data based on plastome sequences have revealed limited amounts of diversity and haplotype sharing among closely related species, suggesting incomplete lineage sorting, gene flow or both (e.g. Hernández-León et al. 2013; Willyard et al. 2016). Plastome-scale datasets are therefore necessary for more accurate phylogenetic hypotheses, analysis of phylogeographical structure and the estimation of gene-flow patterns.

The Mexican pines *Pinus greggii* Engelm. ex Parl., *P. jaliscana* Pérez de la Rosa and *P. oocarpa* Schiede belong to subsection *Australes*, a group comprising approximately 30 species from North and Central America and the Antilles (Gernandt et al. 2005), hypothesized to have undergone a recent radiation (Willyard et al. 2007). A partial plastome sequence has been published for *P. greggii* (Parks et al. 2012), whereas only five plastid DNA markers are available for *P. jaliscana* and *P. oocarpa* (Hernández-León et al. 2013). Detailed phylogenetic relationships among these endemic and recently diverged species have therefore not been addressed. These species are characterized by contrasting ecological and geographical features, from wide to restricted distribution ranges, and from temperate to subtropical environments. *Pinus greggii* is considered Vulnerable and *P. jaliscana* is considered Near Threatened by the International Union for the Conservation of Nature (IUCN 2016) (Supplementary Material 1).

Seeds used in this study were collected within the natural distribution range of each species and voucher specimens were deposited in the Herbario Nacional de México (Supplementary Material 2). Total genomic DNA was extracted from megagametophyte (haploid tissue of maternal origin found in the seed) using a Wizard Genomic DNA Purification kit (Promega, Madison, WI). DNA samples were stored at the Laboratorio Sistemática Molecular Botánica (Instituto de Biología, UNAM, Mexico). Genomic libraries were prepared with 800 ng of DNA, sheared and size-selected to 250 bp (MYcroarray, Ann Arbor, MI). Libraries were sequenced in an Illumina Hi-Seq 2500 with 100 bp paired ends. The pipeline was developed in *P. greggii* for comparing the two *de novo* short-read assemblers, *Velvet* ver. 1.2.10 (Zerbino and Birney 2008) and *SPAdes* ver. 3.6.2 (Bankevich et al. 2012), and then replicated in *P. jaliscana* and *P. oocarpa* using *SPAdes* only. FASTQ files were processed in *Geneious* 9.2.6 (Kearse et al. 2012), removing duplicate reads and trimming low-quality bases.

In the first stage of the pipeline, plastome reads were filtered using the *Geneious* 9.2.6 mapper tool using *P. taeda* NC_021440 as the reference (Liu et al. 2013). Filtered reads were independently assembled *de novo* using *Velvet* ver. 1.2.10 and *SPAdes* ver. 3.6.2. For each independent assembly, *de novo* contigs were mapped over NC_021440 using the *Geneious* Mapper tool with the highest sensitivity option and five iterations for fine tuning. The consensus sequence was generated and segments without any mapped contigs were annotated and filled with *P. taeda* reference sequence to produce a first chimeric draft plastome. In a second stage, the entire read dataset was assembled over the chimeric plastome. Ambiguous calls produced by the assembly of different reads to positions already covered by *de novo* contigs were resolved by selecting the nucleotide displayed by the *de novo* contig. Regions previously filled with *P. taeda* sequence that displayed a poor mapping of reads leading to low coverage (<30×) and/or ambiguous calls were considered gaps. The consensus sequence for this second draft of the plastome was then obtained. In the third stage of the pipeline, we ran the gap filling software *Sealer* (Paulino et al. 2015) for finishing the genome. For details on the parameters used for each step, see Supplementary Material 4.

We compared the two resulting assemblies and retained the sequence based on *SPAdes* as the best version of the *P. greggii* plastome based on number and length of *de novo* contigs (Stage 1) and the number of gaps to be filled during Stages 2 and 3 (Table 1). This result agrees with assays performed on prokaryotic genomes (Magoc et al. 2013).

**Table 1.** General features of the assembly process of the complete plastomes of three Mexican *Pinus* species.

			Stage 1						Stage 2			Stage 3
	Voucher	Reference		Reads mapped to reference and filtered	No. of *de novo* scaffolds (mean length)		No. of scaffolds mapped to reference	No. of gaps (length on	No. of reads mapped to chimeric plastome	Mean	No.	
Species	specimen	plastome	Assembler	(% of dataset)	Total	>200 bp	(mean length)	reference)	(% of dataset)	coverage	of gaps	k^a^
*P. greggii*	DSG1311	NC_021440	Velvet	2,008,556 (22%)	263 (560 bp)		71 (1623 bp)	23 (6303 bp)	1,990,446 (21.8%)	146×	4	90, 80
*P. greggii*	DSG1311	NC_021440	SPAdes	2,008,556 (22%)	180 (1886 bp)		20 (6131 bp)	5 (1375 bp)	1,982,441 (21.7%)	146×	2	90
*P. jaliscana*	DSG456	DSG1311	SPAdes	2,106,522 (32.1%)	1898 (166 bp)	209 (881 bp)	15 (7967 bp)	4 (1373 bp)	2,108,839 (32.1%)	98×	1	90
*P. oocarpa*	DSG711	DSG1311	SPAdes	1,462,364 (18.2%)		36 (3504 bp)	10 (11,871 bp)	6 (1107 bp)	1,814,887 (22.5%)	83×	3	90

Stage 1 corresponds to the *de novo* assembly, Stage 2 to the reference-guided assembly, and Stage 3 to the gap filling step. Final plastome sequences resulted from the pipeline based on *SPAdes*. In *P. jaliscana* and *P. oocarpa*, only scaffolds >200 bp were used for mapping.

^a^ Value of k-mer length where gaps were successfully filled.

The final plastome was annotated by automated transfer of annotations from *P. taeda* NC_021440 based on a 98% similarity criterion (with *Geneious* ver. 9.2.6). Open reading frames (ORFs) were identified and annotations of type ‘gene’ and ‘CDS’ were amended to fit the corresponding ORF when necessary. The inverted repeat regions (IRa, IRb) were transferred from *P. thunbergii* NC_001631 (Wakasugi et al. 1994). Plastome annotation was optimized manually by comparing our plastome to *P. taeda* NC_021440, *P. massoniana* NC_021439 (Huang et al. 2012), *P. thunbergii* NC_001631, *P. tabuliformis* NC_028531 (Peng and Yu 2015) and *P. strobus* NC_026302 (Zhu et al. 2016), all aligned using the MAFFT ver. 7.222 (Katoh et al. 2002) plugin included in *Geneious*, with automatic algorithm selection and default values. Manually edited annotations included *chlL* (lacking a start codon), *trnD-GCA* (corrected to *trnD-GUC*), *rps4* (small version excluded), *trnL-UAA* (orientation corrected), *psaI*, *psbA*, *rpl16*, *ycf1*, *ycf2*, *ycf12*, *trnG* and *trnN* (manually added). Additional details on the manual annotation are found in Supplementary Material 5. The final *P. greggii* plastome sequence comprised 114 genes, 73 coding DNA sequences (CDS), 4 ribosomal RNAs (rRNA) and 36 transfer RNAs (tRNA) genes.

The *P. greggii* whole plastome sequence reported here is 274 bp longer than *P. greggii* partial plastome JN854198 published by Parks et al. (2012), which has 120,227 bp (plus 370 bp as Ns). Main differences of our sequence when compared to JN854198 include 2 insertions (291 bp in *ycf1*, 55 bp in *trnN-chlL*) and 2 deletions (99 bp in *rrn16*, 356 in *cemA-ycf4* characterized by a high proportion of ambiguities). Improvements made on the *P. greggii* plastome can be attributed to methodological differences. For example, Parks et al. (2012) used Illumina 60 to 80 bp single-end lengths vs. 100 bp paired ends here. Plastome JN854198 was based on a 5× coverage for conserved positions and a minimum of 20× for SNPs, while our sequence had a coverage of 146×.

Phylogenetic relationships based on *P. greggii*, *P. jaliscana* and *P. oocarpa* plastomes (GenBank: KY963967, KY963968 and KY963969) were investigated by aligning them to 17 plastomes from *Pinus* subsection *Australes* (Parks et al. 2012) and 2 plastomes from section *Pinus* as an outgroup (Wakasugi et al. 1994; Fang et al. 2016), using MAFFT as previously described. A maximum-likelihood tree was built using RAxML-HPC ver. 8 (Stamatakis 2006) on XSEDE (Miller et al. 2010) with a general time reversible + gamma (GTR + G) nucleotide substitution model and a random stepwise addition parsimony starting tree. Support values were based on 300 rapid bootstrap replicates.

The alignment revealed an 805 bp *psbA* sequence inserted within the *trnK-UUU-matK* region of *P. taeda* NC_021440 (already identified as poorly resolved and considered as a gap during our assembly), which is exclusive of this plastome. Whether this insertion is a characteristic of this species or an inaccuracy remains to be determined. Genes *ycf1* and *ycf2*, despite large differences in size between species (5157–5742 bp and 6090–6210 bp, respectively), were correctly aligned. This is in accordance with previous results showing that these genes harbour a great amount of variability at large and intermediate phylogenetic scales (Parks et al. 2012; Hernández-León et al. 2013).

Phylogenetic analysis supports the existence of a monophyletic Oocarpae (egg-cone pines from Mexico and Central America) and Attenuatae (California closed-cone pines; Figure 1). Within Oocarpae, *P. greggii* (north-eastern Mexico) grouped more closely to *P*. *leiophylla* and *P. chihuahuana* (central and north-western Mexico, respectively). *Pinus jaliscana* (central western Mexico) and *P. oocarpa* (NW Mexico to Nicaragua) were closely related to *P. lawsonii*, *P. pringlei* (SW Mexico) and *P. lumholtzii* (NW Mexico) in general agreement with Hernández-León et al. (2013). Yet, very short internal branch lengths and low bootstrap values hindered a precise determination of sister relationships, suggesting a very recent radiation of Western Mexican pines within *Australes*. Notoriously, our analysis revealed *P. patula* (eastern Mexico) as sister to all the other Mexican species, in contrast to the pattern reported by Parks et al. (2012) where it is sister to the *P. greggii* clade. However, current available *Australes* plastomes from Parks et al. (2012) display a high number of ambiguities (from 120 in *P. lawsonii* to 29,040 in *P. attenuata*), which means that internal branch lengths and phylogenetic relationships should be interpreted cautiously. Future investigations based on fully resolved plastome sequences shall help in building a more detailed understanding of pine evolution in Mexico, which represents the centre of diversity of the genus (Farjon and Filer 2013).

**Figure 1.**
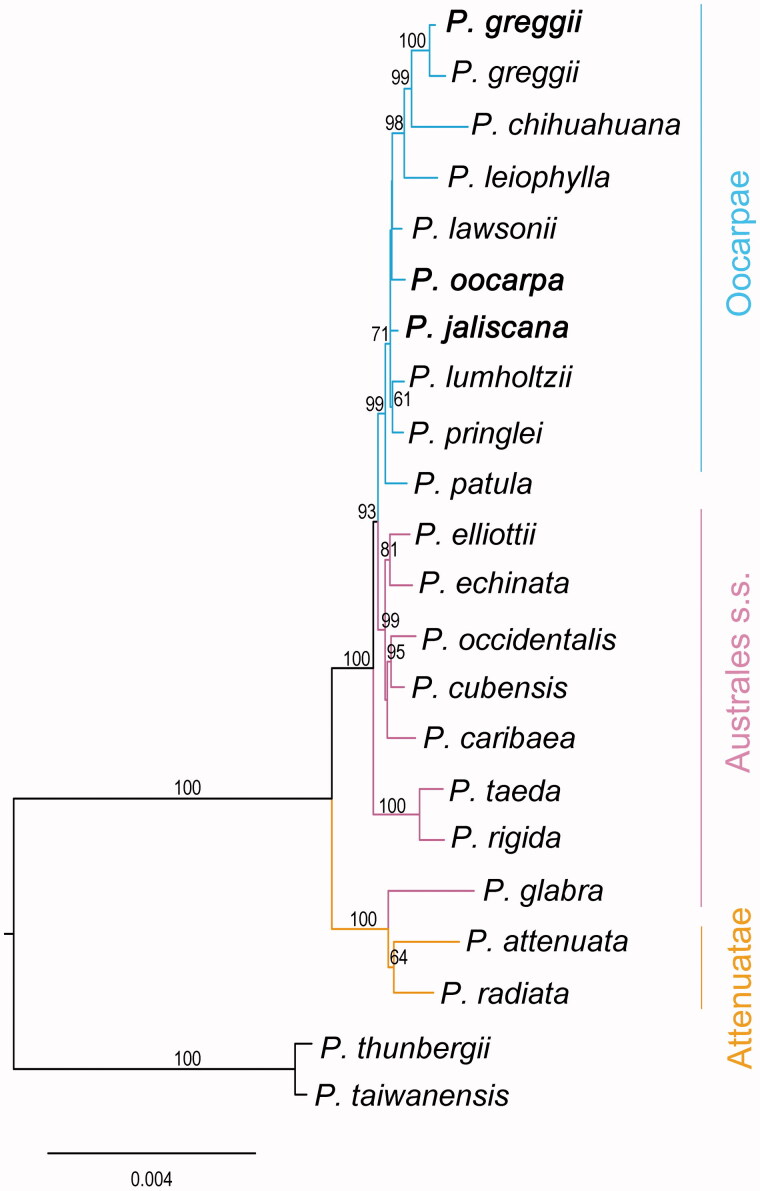
Maximum-likelihood phylogenetic tree among species from *Pinus* subsection *Australes*. Bootstrap values >50% are shown above each branch. Plastomes assembled in the present study are shown in bold. Accession numbers: *P. glabra* JN854199, *P. attenuata* FJ899569, *P. radiata* JN854165, *P. taeda* NC_021440, *P. rigida* JN854163, *P. greggii* KY963967, *P. greggii* JN854198, *P. chihuahuana* FJ899575, *P. leiophylla* JN854187, *P. lawsonii* JN854188, *P. oocarpa* KY963969, *P. jaliscana* KY963968, *P. lumholtzii* JN854186, *P. pringlei* JN854189, *P. patula* JN854175, *P. elliottii* JN854202, *P. echinata* JN854204, *P. occidentalis* JN854177, *P. cubensis* JN854214, *P. caribaea* JN854222, *P. thunbergii* NC_001631, *P. taiwanensis* NC_027415.

## Supplementary Material

TMDN_A_1365637_Supplementary_Information.zip

## Geolocation information

Sierra Madre Oriental and Trans-Mexican Volcanic Belt of Mexico21.052°N, 99.172°W20.357°N, 105.259°W20.882°N, 98.587°W
