# Supplementary material for: Complete plastomes of three endemic Mexican pine species (Pinus subsection Australes)
Source: Mitochondrial DNA B Resour. 2017 Aug 22;2(2):562–5. doi: 10.1080/23802359.2017.1365637 (PMC7799579; doi:10.1080/23802359.2017.1365637)
Supplement: TMDN_A_1365637_Supplementary_Information.zip [file TMDN_A_1365637_SM5538.zip › TMDN_A_1365637_Supplementary_Information.docx]

**ONLINE SUPPLEMENTARY MATERIAL**

Supplementary Material 1. General characteristics of *Pinus* species whose plastomes are assembled in this work, compared to other references available in the GenBank RefSeq database. Species classification in sections and subsections follows Price et al. (1998), area of occurrence and distribution range as described by Farjon & Filer (2013).

| **Species** | **Subgenus** | **Section** | **Subsection** | **Distribution** | | **IUCN status^a^** | **RefSeq** | **Total length** | **Total ambiguities (percentage of length)** | **Annotations** | | | | **Reference** |
| --- | --- | --- | --- | --- | --- | --- | --- | --- | --- | --- | --- | --- | --- | --- |
|  |  |  |  | **Occurrence** | **Range** |  |  |  |  | **Gene** | **CDS** | **tRNAs** | **rRNA** |  |
| *P. greggii* | *Pinus* | *Trifoliae* | *Australes* | NE Mexico | Regional | V |  | 120,501 | 0 | 114^b^ | 73^c^ | 36^d^ | 4 |  |
| *P. jaliscana* | *Pinus* | *Trifoliae* | *Australes* | W Mexico | Restricted | NT |  | 120,715 | 0 | 114 | 73 | 36 | 4 |  |
| *P. oocarpa* | *Pinus* | *Trifoliae* | *Australes* | NW Mexico to Nicaragua | Wide | LC |  | 120,596 | 0 | 114 | 73 | 36 | 4 |  |
| *P. taeda* | *Pinus* | *Trifoliae* | *Australes* | SE United States | Wide | LC | NC_021440.1 | 121,530 | 0 | 111 | 71 | 34 | 4 | Liu et al., 2013 |
| *P. contorta* | *Pinus* | *Trifoliae* | *Contortae* | W Alaska to California and Rocky Mountains | Wide | LC | NC_011153.4 | 120,438 | 5,171 (4.3%) | 110 | 70 | 34 | 4 | Cronn et al., 2008 |
| *P. tabuliformis* | *Pinus* | *Pinus* | *Pinus* | Central to NE China | Wide | LC | NC_028531.1 | 119,646 | 0 | 116 | 74 | 36 | 4 | Peng and Yu 2015 |
| *P. thunbergii* | *Pinus* | *Pinus* | *Pinus* | Japan | Regional | LC | NC_001631.1 | 119,707 | 0 | 171 | 123 | 35 | 4 | Wakasugi et al. 1994 |
| *P. taiwanensis* | *Pinus* | *Pinus* | *Pinus* | Taiwan | Restricted | LC | NC_027415.1 | 119,741 | 0 | 137 | 92 | 36 | 4 | Fang et al., 2016 |
| *P. massoniana* | *Pinus* | *Pinus* | *Pinus* | Central to E China | Wide | LC | NC_021439.1 | 119,739 | 0 | 109 | 73 | 36 | 0 | Huang et al. 2013 |
| *P. bungeana* | *Strobus* | *Quinquefoliae* | *Gerardianae* | N-central China | Wide | LC | NC_028421.1 | 117,861 | 1 | 113 | 71 | 36 | 4 | Li et al., 2016a |
| *P. gerardiana* | *Strobus* | *Quinquefoliae* | *Gerardianae* | Northwestern Himalayas | Wide | NT | NC_011154.4 | 117,618 | 2,152 (1.8%) | 110 | 70 | 34 | 4 | Cronn et al., 2008 |
| *P. koraiensis* | *Strobus* | *Quinquefoliae* | *Strobus* | Around the Sea of Japan | Regional | LC | NC_004677.2 | 117,190 | 0 | 315 | 273 | 36 | 4 | Noh et al., 2010 |
| *P. armandii* | *Strobus* | *Quinquefoliae* | *Strobus* | NW to Central China, Taiwan, Myanmar | Wide | LC | NC_029847.1 | 117,265 | 0 | 115 | 74 | 36 | 4 | Li et al., 2016b |
| *P. lambertiana* | *Strobus* | *Quinquefoliae* | *Strobus* | W American Pacific coast, Oregon to Baja California | Wide | LC | NC_011156.4 | 117,239 | 1,713 (1.5%) | 110 | 71 | 33 | 4 | Cronn et al,. 2008 |
| *P. sibirica* | *Strobus* | *Quinquefoliae* | *Strobus* | N-Central Asia, Kazakhstan to Siberia | Wide | LC | NC_028552.2 | 116,635 | 0 | 113 | 81 | 28 | 4 | Baturina et al., 2016 |
| *P. strobus* | *Strobus* | *Quinquefoliae* | *Strobus* | NE United States, and S Mexico | Wide | LC | NC_026302.1 | 115,576 | 0 | 111 | 70 | 35 | 4 | Zhu et al., 2016 |
| *P. krempfii* | *Strobus* | *Quinquefoliae* | *Krempfianae* | S Vietnam | Restricted | V | NC_011155.4 | 116,989 | 2,427 (2.1%) | 108 | 69 | 32 | 4 | Cronn et al., 2008 |
| *P. longaeva* | *Strobus* | *Parrya* | *Balfourianae* | E California to Colorado Plateau | Regional | LC | NC_011157.3 | 117,726 | 2,928 (2.5%) | - | - | - | - | Cronn et al., 2008 |
| *P. monophylla* | *Strobus* | *Parrya* | *Cembroides* | SW United States to Baja California | Wide | LC | NC_011158.4 | 116,479 | 2,648 (2.3%) | 111 | 70 | 34 | 4 | Cronn et al., 2008 |
| *P. nelsonii* | *Strobus* | *Parrya* | *Nelsoniae* | NE Mexico | Restricted | X | NC_011159.4 | 116,834 | 3,452 (3%) | 111 | 34 | 70 | 4 | Cronn et al., 2008 |

^a^ LC: least concern, NT: near threatened, V: vulnerable.

^b^Additional gene annotations when compared to *P. taeda* NC_021440 include *ycf12*, *psaM*, *trnG*, *trnN*; annotations that were removed include *rps4*.

^c^ Additional CDS annotations when compared to *P. taeda* NC_021440 include *ycf12*, *psaM, psbA*; *chlL* was removed.

^d^ Additional tRNA annotations when compared to *P. taeda* NC_021440 include *trnG* and *trnN*.

**References**

Baturina OA, Tupikin AE, Petrova EA, Goroshkevich SN, Kabilov MR. 2016. *Pinus sibirica* chloroplast, complete genome. NCBI Reference Sequence: NC_028552.2

Cronn R, Liston A, Parks M, Gernandt DS, Shen R, Mockler T. 2008. Multiplex sequencing of plant chloroplast genomes using Solexa Sequencing-by-synthesis Technology. Nucleic Acids Res 36:e122.

Fang MF, Wang YJ, Zu YM, Dong WL, Wang RN, Deng TT, Li ZH. 2016. The complete chloroplast genome of the Taiwan red pine *Pinus taiwanensis* (Pinaceae). Mitochondrial DNA 27:2732-2733.

Farjon A, Filer D. 2013. An Atlas of the World’s Conifers. Boston: Brill.

Huang SW, Liu TY, Zhou F, Liu CX, Luo M, Cai YL, Wang XN, Chen JH. 2012. *Pinus massoniana* chloroplast, complete genome. NCBI Reference Sequence: NC_021439.1

Li ZH, Qian ZQ, Liu ZL, Deng TT, Zu YM, Zhao P, Zhao GF. 2016b. The complete chloroplast genome of Armand pine *Pinus armandii* an endemic conifer tree species to China. Mitochondrial DNA 27:2635-2636.

Li ZH, Zhu J, Yang YX, Yang J, He JW, Zhao GF. 2016a. The complete plastid genome of Bunge's pine *Pinus bungeana* (Pinaceae). Mitochondrial DNA 27:2971-2972.

Liu TY, Huang SW, Li QF, Wang JB, Liu CX, Ning HG, Zhou HB, Chen JH. 2013. *Pinus taeda* chloroplast complete genome. NCBI reference sequence: NC_021440.

Noh EW, Lee JS, Choi YI, Han MS, Yi YS, Han SU. 2010. *Pinus koraiensis* chloroplast, complete genome. NCBI Reference Sequence: NC_004677.2

Peng S, Yu Z. 2015. *Pinus tabuliformis* voucher PTAB20150818V5 chloroplast, complete genome. NCBI Reference Sequence: NC_028531.1

Price RA, Liston A, Strauss SH. 1998. Phylogeny and systematics of *Pinus*. In: Richardson DM, editor. Ecol Biogeogr *Pinus*. Cambridge: Cambridge University Press; p. 49–68.

Wakasugi T, Tsudzuki J, Ito S, Nakashima K, Tsudzuki T, Sugiura M. 1994. Loss of all ndh genes as determined by sequencing the entire chloroplast genome of the black pine Pinus thunbergii. Proc Natl Acad Sci U S A. 91:9794–9798.

Zhu A, Guo W, Gupta S, Fan W, Mower JP. 2016. Evolutionary dynamics of the plastid inverted repeat: The effects of expansion, contraction, and loss on substitution rates. New Phytol 209:1747.

**Supplementary Material 2.** Passport data of the voucher specimens deposited in herbarium MEXU.

| **Species** | **Collection Number** | **Date** | **Country** | **State** | **County** | **Locality** | **Coordinates** | | **Altitude (masl)** | **Collectors** |
| --- | --- | --- | --- | --- | --- | --- | --- | --- | --- | --- |
|  |  |  |  |  |  |  | **N** | **W** |  |  |
| *Pinus greggii* | DSG1311 | 17/05/2015 | Mexico | Hidalgo | Jacala de Ledezma | Laguna Seca | 21.052107 | 99.172356 | 1752 | DS Gernandt & S. Salinas López |
| *Pinus jaliscana* | DSG456 | 15/05/2004 | Mexico | Jalisco | Cabos Corrientes | El Tuito, gravel road leading to minas de Zimapan | 20.357222 | 105.259722 | 1093 | DS Gernandt, M González Ledesma, JA Perez de la Rosa, G Vargas Amado |
| *Pinus oocarpa* | DSG711 | 25/08/2006 | Mexico | Hidalgo | Calnali | Calnali, Cerro de la Aguja (Huizmaloteptl) | 20.882222 | 98.5875 | 1215 | DS Gernandt, M González Ledesma |

**Supplementary Material 3.** Parameters used in the programs used for the assembly of *P. greggii* plastome

Plastome reads filtering: *Geneious* 9.2.6 mapper tool with Medium-Low sensitivity and 5 iterations, keeping all the used reads (including the mates of any assembled reads, even if the mate wouldn’t be assembled).

Stage 1: *Velvet* short read assembler ver. 1.2.10 with the following parameters: k-mer length 35, low-coverage cutoff 5, high-coverage cutoff 200, and a minimum contig length of 100 bp. Expected coverage and insert size were internally calculated by *Velvet*. The *de novo* contigs were mapped to *P. taeda* using the *Geneious* mapper with Medium Sensitivity and 5 iterations. The consensus sequence was generated by using the Highest Quality threshold and assigning Total Quality to the consensus nucleotides (i.e., the sum of contributing bases minus non-contributing bases).

Stage 2: Entire reads dataset assembled over draft plastome using the *Geneious* mapper with Medium-Low Sensitivity and 5 iterations for fine tuning.

Stage 3: Gap-filling software *Sealer*. This tool first identifies Ns in the scaffold (total number of Ns does not need to represent gap length) and extracts 100 bp of flanking nucleotides both upstream and downstream of each gap. Given a k-mer length, the gap-flanking sequence pairs are used to build a Bloom filter employed to perform a bidirectional graph search of a path connecting the flanking sequences, using as input a set of reads representing the original dataset from which the draft assembly is generated. We ran *Sealer* with the draft plastome sequence and the entire reads dataset as inputs, without pre-built Bloom filters, allowing for a maximum number of 10 paths using 21 values of k-mers from 95 to 75 bp (options -b20G -P10 -k95 to -k75).

**Supplementary Material 4.** Additional details related to the assembly process of *P. jaliscana* and *P. oocarpa* plastomes.

**Assembly of *Pinus jaliscana* DSG456 plastome**

Plastome dataset: Generated with the same parameters previously described, but using as reference *P. greggii* DSG1311.

Stage 1: Assembly was performed by following pipeline 2 based on the assembler SPAdes. Only scaffolds >200 bp were retained for mapping. Subsequent steps and annotation were accomplished following the same methods previously described. The final plastome DSG456 was aligned to *P. greggii* DSG1311 using the MAFFT v. 7.222 plugin included in *Geneious*, with automatic algorithm selection and default values in order to verify all transferred annotations and detect potential inconsistencies worth of manual revision.

**Assembly of *Pinus oocarpa* DSG711 plastome**

Plastome dataset: We used as reference *P. greggii* DSG1311. Usage of the same parameters previously applied to *P. greggii* and *P. jaliscana* led to a large number of contigs produced in the *de novo* assembly step and a high amount of ambiguous calls when such scaffolds were mapped. The stringency of mapping conditions was therefore increased by mapping them to DSG1311 reference with Custom sensitivity, with the following parameters: read’s minimum mapping quality 30, only map paired reads which map nearby, minimum overlap between the read and the reference sequence 65 bp and minimum overlap identity of 95%, accurately map reads with errors to repeat regions, maximum number of mismatches per read 8%. Gaps were allowed with a maximum of 10% of read length. Indexing options were set as follows: word length 30, index word length 13, ignore words repeated more than 8 times.

Stage 1: The filtered reads were assembled using the *de novo* assembler SPAdes with the same parameters as previously described. Only scaffolds >200 bp were retained and mapped to DSG1311. Subsequent steps and annotation were accomplished following the same methods previously described. The final plastome DSG711 was aligned to *P. greggii* DSG1311 using the MAFFT v. 7.222 plugin included in *Geneious*, with automatic algorithm selection and default values in order to verify all transferred annotations and detect potential inconsistencies worth of manual revision.

**Supplementary Material 5.** Details on the manual annotation of 11 genes based on the alignment of *Pinus greggii* plastome to four additional *Pinus* references.

The *chlL* gene annotation was successfully transferred from NC_021440, but no ORF was associated with it. We identified that the expected ATG start codon was replaced by a GTG codon. In consequence, we added a note on the *chlL* gene annotation for specifying the lack of the first A nucleotide and the CDS annotation was removed.

The *trnD-GCA* gene was found in the same position, with the same sequence and under the same name as in reference plastomes from *P. taeda*, *P. tabuliformis* *P. massoniana,* and *P. thunbergii*. However, this gene was annotated as *trnD-GUC* in the *P. strobus* reference plastome*.* We therefore verified the identity of this tRNA gene by performing a BLAST search (Altschul et al. 1997) of the 74 bp sequence in the GtRNAdb database of eukaryotic tRNAs (Chan & Lowe 2009). The most similar matches (E-value ≤4x10^-22^) included fourteen sequences that corresponded to *trnD-GUC* genes. Moreover, the *GCA* anticodon corresponds to the aminoacid cysteine (C), not aspartate (D). The gene annotation was therefore corrected to *trnD-GUC*.

The *psaI* gene was not transferred in the first annotation step, and was added manually with a 96.3% similarity due to the duplication of a 12 bp motif relative to the *psaI* gene from *P. taeda* NC_021440. Accordingly, the annotation length was increased by 12 bp to match the corresponding ORF.

The *psbA* gene (81 bp) was successfully transferred from NC_021440, but was lacking a CDS annotation. The corresponding CDS annotation was transferred from *P. sibirica* NC_028552 with a 98.77% similarity.

The *rpl16* gene annotation was successfully transferred but the corresponding CDS annotation (with 99.75% similarity to *P. greggii*) was lacking. We manually added it at the expected position.

*Pinus taeda* NC_021440 had an additional 249 bp *rps4* annotation between *trnT-GGU* and *psaM*. This annotation represented the middle portion of the 606 bp *rps4* gene located 19,157 bp downstream. Given that this annotation was not transferred to *P. greggii* in our first annotation step, that it represents a smaller portion of the larger *rps4* and does not correspond to any ORF at this position, we did not include it.

The *trnL-UAA* gene was annotated in the forward direction in *P. taeda* NC_021440, while it had a reverse orientation in *P. thunbergii* NC_001631 and *P. strobus* NC_026302. We retained the reverse orientation based on the sequence and secondary structure reported for *trnL-UAA* (Taberlet et al. 2007).

The genes *ycf1* and *ycf2* were not transferred in the first automated step due to the presence of nucleotide substitutions and a different configuration of repetitive elements. They were therefore transferred manually with a 79.9% and 66.6% similarity, respectively.

The *ycf12* gene and CDS annotations were absent from *P. taeda* and *P. thunbergii* reference plastomes, but present in *P. tabuliformis*, *P. massoniana* and *P. strobus*. Given that the 102 bp gene sequence was present in *P. greggii* with a 100% similarity, we transferred the annotation manually.

Two additional tRNAs from *P. thunbergii* (*trnG* and *trnN*) not present in *P. taeda* NC_021440 were found in *P. greggii* with 100% similarity. We transferred the corresponding annotations manually, increasing the number of tRNAs from 34 to 36.

**References**

Altschul SF, Madden TL, Schaffer AA, Zhang J, Zhang Z, Miller W, Lipman DJ. 1997. Gapped BLAST and PSI-BLAST: a new generation of protein database search programs. Nucleic Acids Res 25:3389.

Chan PP, Lowe TM. 2009. GtRNAdb: A database of transfer RNA genes detected in genomic sequence. Nucleic Acids Res 37(Database issue):D93.

Taberlet, P., Coissac, E., Pompanon, F., Gielly, L., Miquel, C., Valentini, A., Vermat, T., Corthier, G. Brochmann, C., Willerslev, E. 2007. Power and limitations of the trnL(UAA) intron for plant DNA barcoding. Nucleic Acids Res 35:e14.
